# MET and RON receptor tyrosine kinases in colorectal adenocarcinoma: molecular features as drug targets and antibody-drug conjugates for therapy

**DOI:** 10.1186/s13046-020-01711-x

**Published:** 2020-09-22

**Authors:** Hang-Ping Yao, Xiang-Min Tong, Rachel Hudson, Ming-Hai Wang

**Affiliations:** 1 State Key Laboratory for Diagnosis and Treatment of Infectious Diseases, First Affiliated Hospital, Zhejiang University School of Medicine, Hangzhou, China; 2 National Clinical Research Center for Infectious Diseases, Zhejiang University School of Medicine, Hangzhou, China; 3 Department of Hematology, Zhejiang Provincial People’s Hospital, People’s Hospital of Hangzhou Medical College, Hangzhou, China; 4 Cancer Biology Research Center, Texas Tech University Health Sciences Center, Amarillo, USA; 5 Department of Pharmaceutical Sciences, School of Pharmacy, Texas Tech University Health Sciences Center, TX Amarillo, USA

**Keywords:** Colorectal cancer, Receptor tyrosine kinase, Tumorigenesis, Therapeutic monoclonal antibody, Dual targeting antibody, Antibody-drug conjugates, Pharmaceutic efficacy, Pharmacokinetics, Toxicology, Clinical trials

## Abstract

Advanced colorectal adenocarcinoma (CRAC), featured by distinctive histopathological appearance, distant organ metastasis, acquired chemoresistance, and tumorigenic stemness is a group of heterogeneous cancers with unique genetic signatures and malignant phenotypes. Treatment of CRAC is a daunting task for oncologists. Currently, various strategies including molecular targeting using therapeutic monoclonal antibodies, small molecule kinase inhibitors and immunoregulatory checkpoint therapy have been applied to combat this deadly disease. However, these therapeutic modalities and approaches achieve only limited success. Thus, there is a pharmaceutical need to discover new targets and develop novel therapeutics for CRAC therapy. MET and RON receptor tyrosine kinases have been implicated in CRAC pathogenesis. Clinical studies have revealed that aberrant MET and/or RON expression and signaling are critical in regulating CRAC progression and malignant phenotypes. Increased MET and/or RON expression also has prognostic value for CRAC progression and patient survival. These features provide the rationale to target MET and RON for clinical CRAC intervention. At present, the use of small molecule kinase inhibitors targeting MET for CRAC treatment has achieved significant progress with several approvals for clinical application. Nevertheless, antibody-based biotherapeutics, although under clinical trials for more than 8 years, have made very little progress. In this review, we discuss the importance of MET and/or RON in CRAC tumorigenesis and development of anti-MET, anti-RON, and MET and RON-dual targeting antibody-drug conjugates for clinical application. The findings from both preclinical studies and clinical trials highlight the potential of this novel type of biotherapeutics for CRAC therapy in the future.

## Background

Advanced colorectal adenocarcinoma (CRAC), defined by its histopathological appearances, metastatic dissemination, and acquired chemoresistance, is a distinctive, heterogenic and aggressive cancer [1, 2]. During the last decade, genetic aberrations and cellular disorganizations associated with CRAC initiation, progression, and malignancy have been extensively studied using various platforms such as genetic, transcriptomic, proteomic, epigenomic, and tumor microenvironmental approaches [1–4]. The findings from these analyses establish a unique genetic profile featuring sequential accumulation of mutations in adenomatous polyposis coli (APC), reticular activating system (RAS), tumor suppressor protein 53 (TP53), and small mothers against decapentaplegic 4 (SMAD4) genes and aberrant signaling pathways associated with Wnt/β-catenin, human epidermal growth factor receptor (HER) family, and transforming growth factor (TGF)-β/SMADs [1–4]. Moreover, activating mutation in v-Raf murine sarcoma viral oncogene homolog B (BRAF) and inactivation of the DNA mismatch repair gene have also been involved in CRAC development [5, 6]. Recently, molecular subtyping has established a consensus molecular classification of CRAC into four consensus molecular subtypes [7, 8]. These discoveries significantly help us understand the nature of CRAC heterogeneity associated with malignancy and lay the foundation for clinical stratification and subtype-based targeted intervention.

**Fig. 1:**
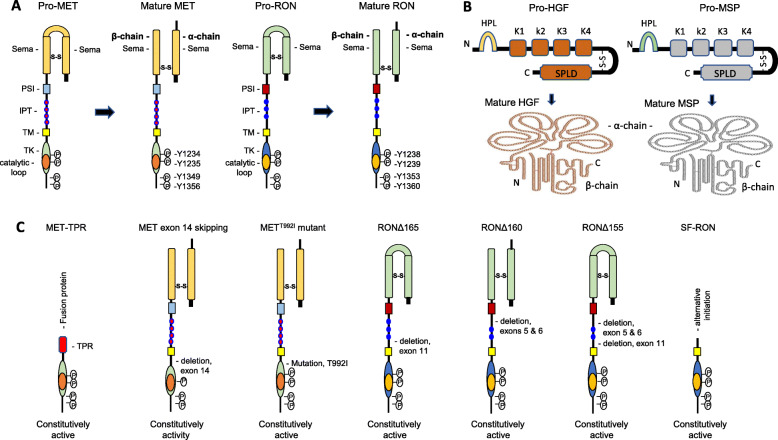
Schematic representation of MET and RON, their ligands HGF and MSP, and representative isoforms. (**a**)Both MET and RON are first synthesized as a biologically inactive single-chain precursor (pro-MET and pro-RON). Mature MET is composed of a 45 KDa α-chain and a 145 kDa β-chain linked by a disulfide bound. Similarly, mature RON consists of a 40 kDa α-chain linked through a disulfide bond to a 145 kDa β-chain. Structurally, both MET and RON consist of a large extracellular domain, a short transmembrane (TM) segment, and a cytoplasmic portion harboring a tyrosine kinase (TK) domain and a C-terminal tail. The β-chain of MET and RON contains a large portion of the semaphorin (SEMA) domain followed by a plexin-semaphorin-integrin (PSI) domain and 3 or 4 immunoglobulin-like plexin and transcription (IPT) motifs. Regulatory tyrosine residues, Tyr^1334^ and Tyr^1335^ in the MET TK domain and Tyr^1238^ and Tyr^1239^ in the RON TK domain, are indicated. Also, Tyr^1349^ and Tyr^1356^ in the MET C-terminal tail and Tyr^1353^ and Tyr^1360^ in the RON C-terminal tail, which form the functional docking site, respectively, are marked. (**b**) Both HGF and MSP are first synthesized as biologically inactive single-chain precursors known as pro-HGF and pro-MSP. Proteolytic cleavage results in a biologically active two-chain form of mature HGF and MSP. Both α-chains of HGF and MSP contains a hairpin loop (HPL) followed by four kringle domains (K1 to K4). Both β-chains of HGF and MSP contain a serine protease-like domain (SPLD) with substation of amino acids in the active site. The high affinity MET-binding site is in the HGF α-chain and the low affinity MET-binding site is in the HGF β-chain. In contrast, the major RON-binding site is in the MSP β-chain and the minor RON-binding site is in the MSP α-chain. (**c**) Representative MET isoforms and RON variants are presented. MET-TPR is a 65 KDa fusion protein generated by a chromosomal rearrangement between the *translocated promoter region* (TPR) and the MET intracellular sequence containing the kinase domain and the C-terminal tail. MET^T992I^ mutant is a constitutively active isoform identified in CRAC samples. MET exon-14 skipping variant is produced by aberrant splicing due to mutations leading to exon 14 skipping. This variant is unable to interact with the E3 ubiquitin-protein ligase CBL leading to impaired MET degradation with enhanced tumorigenic activity. Splicing variants of RON include RONΔ165 with a deletion of exon 11; RONΔ160 with a combined deletion of exons 5 and 6; RONΔ155 with a combined deletion of exons 5, 6, and 11; and short form (SF) RON, which is initiated by an alternative promoter in the RON gene

Non-surgical treatment of CRAC aimed at prolonging survival is a serious clinical challenge. Except first-line chemotherapies, molecularly targeted and immunoregulatory approaches have been applied with clinical benefits [1, 2, 9, 10]. Nevertheless, there is still a hug gap to achieve the curative outcome for the majority of CRAC patients. Mesenchymal-epithelial transition (MET) and recepteur d’Origine nantais (RON) belong to a unique subfamily of receptor tyrosine kinases (RTK) (Fig. 1) [11, 12]. Since their discovery in 1987 [13] and 1993 [14], respectively, roles of MET and RON in CRAC have been extensively studied, which demonstrate their importance in tumor progression, malignancy, and stemness [11, 12]. Also, therapeutics such as small molecule kinase inhibitors (SMKIs) and therapeutic monoclonal antibodies (TMABs) targeting MET and/or RON have been validated in preclinical studies and clinical trials [15, 16]. Several SMKIs such as crizotinib and capmatinib, have been approved for clinical application [17, 18]. Nevertheless, TMABs targeting MET and/or RON have made little progress in clinical CRAC application. Up to now, no anti-MET TMABs have been approved by the FDA. This hampers the development of TMAB-based biotherapeutics targeting MET and/or RON for CRAC treatment. Here we present the latest evidence of MET and RON in CRAC pathogenesis and provide evidence of using antibody-drug conjugates (ADC) targeting MET, RON, or both receptors for CRAC therapy. Considering their pharmaceutical properties, therapeutic efficacy, and toxicological profiles [15, 16], MET and RON-targeted ADC therapy should be considered as a promising CRAC treatment option in the future.

### MET and RON in CRAC Pathogenesis

For the last 30 years, roles of MET and RON in CRAC have been established [11, 12]. Currently, genetic evidence supporting MET and/or RON as causative agents that initiate CRAC has not been documented. In animal models, disruption of epithelial MET expression causes significant reduction in numbers of colorectal adenoma [19, 20]. Also, transgenic hepatocyte growth factor (HGF) expression results in increased growth of colorectal xenograft tumors [21, 22]. Evidence that MET activation promotes CRAC cell metastasis in knockout mice has also been demonstrated [21, 22]. In this case, MET signaling facilitates CRAC cells to adhere to liver sinusoidal endothelial cells [21, 22]. These findings indicate that the HGF-MET signaling axis participates in different stages of the colorectal tumorigenic progress. Studies of colon adenoma development have shown that RON may be important in normal colorectal tissue homeostasis, but its expression is not required for the formation and growth of adenoma associated with APC mutation [23]. Instead, RON is involved in regulating the CRAC malignant phenotype, facilitating CRAC cell growth, invasion, and chemoresistance [24–27].

Genetic alterations in MET and RON genes are characterized by activating mutation, gene amplification, and/or aberrant exon splicing (Fig. 1) [12, 28, 29]. An activating mutation in the kinase domain (T992I) of MET has been identified in ~ 5% of primary CRAC cases [30]. CRAC cells harboring this mutation have increased invasive activity [30]. MET gene amplification exists in primary CRAC samples with variable prevalence ranging from 0.5 to 18% [31–34]. Aberrant MET splicing variant with exon 14 skipping has also been reported in certain CRAC cases (Fig. 1c) [35, 36]. The exon 14 skipping results in a MET isoform with increased oncogenic activity, which appears to act as a driving force for CRAC malignancy [35, 36]. In contrast, genetic mutation and gene amplification are rarely observed in the RON gene. There is no report showing activating mutations in the RON gene in CRAC samples. However, various RON splicing isoforms with different exon deletions have been observed in CRAC samples (Fig. 1c) [25, 37, 38]. These isoforms are generated by alternative mRNA splicing, not by the gene mutation [25, 37, 38]. Thus, MET and RON are tumorigenic determinants that predominantly regulate CRAC tumorigenic activity and malignant phenotype.

**Fig. 2:**
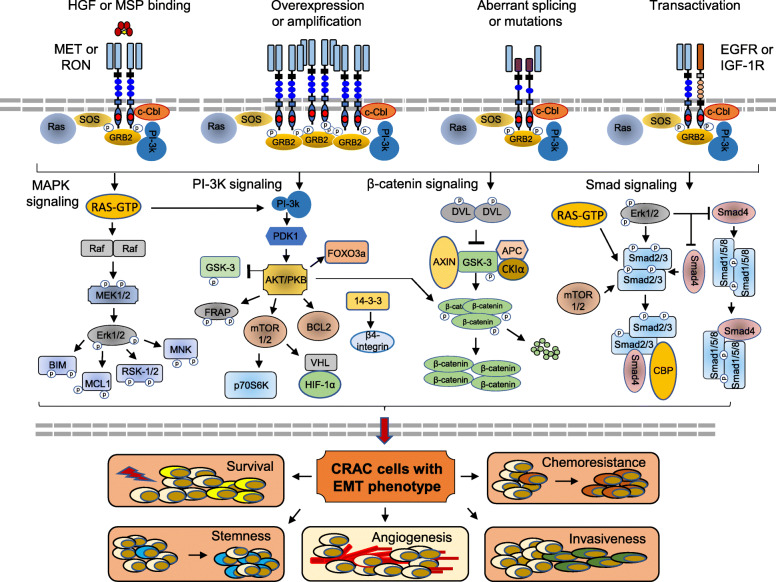
Mechanisms of MET and/or RON activation, signaling pathway, and biological consequence. Activation of MET and/or RON in CRAC cells, in general, is mediated through five events including ligand binding, activating mutation, receptor overexpression, aberrant splicing/alternative initiation, and transactivation through other RTKs such as EGFR and IGF-1R. HGF or MSP-induced MET and/or RON activation, a classical model, is functional through phosphorylation of several critical tyrosine residues and creates the C-terminal functional docking site, which recruits cytoplasmic molecules such as SOS and GRB2. The negative modulator c-CBL, a ubiquitin ligase, also binds the docking site and mediates MET and/or RON endocytosis and degradation. Multiple signaling pathways, such as RAS/MAP kinase, PI3K/AKT, Wnt/β-catenin, and TGF-β/SMAD pathways are activated upon MET and/or RON phosphorylation in CRAC cells, which creates a complex intracellular signaling network. The biological consequence is induction of EMT in CRAC cells leading to increased cellular survival, invasiveness, chemoresistance, and tumorigenic stemness. Briefly, activation of the RAS/MAP kinase cascade stimulates MET and/or RON-mediated activities such as cellular survival, invasiveness, chemoresistance, and tumorigenic stemness through regulating various gene expressions and cellular activities. Activated Erk1/2 also stimulates RSK-2, which regulates not only gene transcription but also cytoskeleton re-organization to cause the EMT-like phenotype. The PI3K-AKT pathway is essential in MET and/or RON-mediated cellular invasive growth and chemoresistance. Activated AKT inhibits GSK-3β by phosphorylation, resulting in MET and/or RON signaling cross-talking with the β-catenin pathway. AKT signaling is also linked to MET and/or RON-induced mTOR phosphorylation, which releases HIF-1α from the VHL. Similarly, mTOR stimulates p70S6 kinase, which activates certain transcription factors leading to increased gene expression. AKT also stimulates 14-3-3 phosphorylation, which displaces α6β4 integrin from its location at hemidesmosomes and re-localizes it to lamellipodia for cell motility. MET and/or RON activation also collaborates with TGF-β mediated Smad2/3 signaling and regulates CRAC cell EMT-like phenotypes, leading to cellular senescence, migration, and chemoresistance. Studies also show that MET and/or RON activation regulate β-catenin dephosphorylation by activating DVL, leading to β-catenin accumulation and nuclear translocation for activating gene transcription. ABL, Abelson murine leukemia viral oncogene homolog; AKT, BCL-2, B cell lymphoma-2; BIM, Bcl-2-like protein 11; CBL, protein kinase B; APC, adenomatous polyposis coli; AXIN, axis inhibition protein; CBP, CREB-binding protein; CREB, cAMP response element-binding protein; DVL, disheveled; Erk, extracellular signal-regulated kinase; FRAP, FKBP12-rapamycin-associated protein; FOXO3a, forkhead box O3; GRB2, growth factor receptor-bound protein; GSK, glycogen synthase kinase; MCL-1, myeloid cell leukemia 1; MEK, mitogen-activated protein kinase-kinase; MNK, mitogen-activated protein kinase interacting protein; mTOR, mammalian target of rapamycin; P70S6K, rribosomal protein S6 kinase beta-1; PDK1, 3-phosphoinositide-dependent protein kinase-1; PI3K, phosphatidyl-inositol 3 kinase; Raf, rapidly accelerated fibrosarcoma; RAS, reticular activating system; RSK-2, p90 ribosomal S6 kinase-2; Smad, small mothers against decapentaplegic; SOS, son of sevenless; TGF, transforming growth factor; VHL, von Hippel-Lindau protein

Pathogenesis of MET and/or RON in CRAC is characterized by phenotypic plasticity known as epithelial to mesenchymal transition (EMT), featuring increased survival, invasive growth, acquired chemoresistance, and tumorigenic stemness (Fig. 2) [11, 12, 22, 24–27, 30–38]. EMT in CRAC cells appears to be regulated at least by dysregulated metabolic, Wnt/β/catenin, RTKs, and stemness pathways [2, 39–41]. The role of MET and/or RON in regulating the metabolic process in CRAC cells is currently unknown. However, both MET and RON, beside stimulation of the MAP kinase pathway, are capable of activating the Wnt/β-catenin pathway, which is critical for colonic epithelial cell transformation, invasiveness, and stemness [11, 12]. Another pathway activated by MET and RON is PI3K signaling, which is vital in both receptor-mediated invasiveness and chemoresistance (Fig. 2) [26, 42]. As a member of the RTK family, aberrant MET and/or RON expression is a common pathological feature in CRAC [11, 12]. In addition, both ligand-dependent and independent activation of MET and/or RON exist in CRAC (Fig. 2). Accumulated evidence further demonstrates that MET and/or RON expression and signaling are sustained in stem-like tumor-initiating cells in CRAC [19, 43], which regulates tumorigenic stemness through an autocrine/paracrine canonical β catenin signaling loop [19, 43], and facilitates stem-like phenotypic transition towards EMT [11, 12]. Thus, aberrant MET and RON expression and signaling play a unique role in regulating and controlling plasticity of CRAC cells towards metastasis and chemoresistance.

Clinical studies using immunohistochemical (IHC) staining provide insight into the status of MET and/or RON expression in primary CRAC samples. In the majority of IHC studies using primary CRAC samples, high frequencies of MET and/or RON expression are documented [25, 44–54]. MET is positive in more than 70% of primary CRAC samples with overexpression in ~ 35% of cases [44–52]. RON is expressed in more than 80% of primary CRAC samples with overexpression in ~ 40% of cases [25, 53, 54]. Small scale IHC analysis using different stages of CRAC samples suggest a trend of MET and/or RON expression increasing from normal to adenoma to adenocarcinoma, and finally to metastatic lesions [25, 44, 45]. In these cases, increased MET and/or RON expression is likely to be linked with gene amplification, abnormal protein accumulation, or both. The patterns of IHC staining for both MET and RON are similar with predominant membrane, predominant cytoplasm, and mixed staining appearances [25, 44–54]. This expression pattern is unlikely to be related to a particular subtype of CRAC, implying that increased MET and/or RON expression is a random event occurring in different subtypes of CRAC.

Clinically, increased expression of MET, RON, or both receptors has been shown to have prognostic value [25, 44–54]. For instance, increased MET expression is statistically different in advanced stages of CRAC with overall survival and cancer-related mortality rates [44–54]. However, association between MET expression and recurrence or disease-free interval is not observed [45]. Other reports show that increased MET or RON expression is associated with shortened overall survival and progression-free survival of CRAC patients [48, 51]. Moreover, the risk of tumor recurrence in patients with overexpression of both receptors is approximately 11 times greater than for patients showing low levels of MET and RON expression [48]. Nevertheless, certain studies did not find correlations between increased MET expression and disease recurrence, survival, or distant metastasis, although MET is highly expressed in the majority of CRAC cases [46]. Considering differences in IHC staining using different antibodies and criteria for judging MET and/or RON expression, a standardized IHC method is definitely needed to clarify discrepancies observed in these studies. Regardless of these observations, IHC staining provides valuable clues about the status of MET and RON expression in CRAC and highlights potentials of using these two receptors as biomarkers for clinical prognosis.

### MET and RON as CRAC Pharmaceutical Targets

Tumorigenesis of MET and RON in CRAC provide the rationale to target them for clinical application. Pharmaceutically, SMKIs and TMABs specific to MET and/or RON have been evaluated in preclinical and clinical settings [11, 12, 16, 28, 29]. For the scope of this review, we will focus on development of anti-MET TMAB-based biotherapeutics, especially antibody-drug conjugates (ADC). Several features of MET and RON as CRAC targets are worth mentioning. **First**, MET and RON are highly and preferentially expressed in CRAC cells but minimal in their corresponding normal epithelial tissues [25, 44–54]. This notion is supported by results from various IHC staining as described above, which renders CRAC as an ideal type of cancer using MET and RON-targeted biotherapeutics for clinical intervention. **Second**, both MET and RON expressed by CRAC cells are highly sensitive to antibody-induced internalization (Table 1). For ADCs to be effective, antibody-induced robust receptor internalization is essential to deliver sufficient payloads for cancer cell killing [55, 56]. **Third**, targeted delivery of payloads by anti-MET and/or RON mAbs is highly effective *in vitro* in killing CRAC cells [57–63]. This effect is proportionally correlated with levels of MET and/or RON expression by CRAC cells. The minimal levels of receptor expression required to achieve more than 95% of killing are about 100,000 MET molecules per cell and 10,000 RON molecules per cell [57–61]. In addition, the approach of using antibody-directed drug delivery overcomes the shortcomings that have occurred in SMKI- and TMAB-targeted CRAC therapy, which rely on addiction of MET and/or RON signaling for cellular survival [11, 12, 15]. **Finally**, studies from animal models have proven that anti-MET and anti-RON mAb-directed drug delivery inhibits CRAC xenograft growth, although the efficacy varies significantly [57, 63].

**Table 1:** Biochemical and biological features of various ADCs specific to MET, RON, and both receptors in different stages of development^a^

**Names of ADCs in development **	**Produced by institutions **	**mAbs, target, IgG subtype **	**Linker properties **	**mAb binding affinity **	**Receptor internalization **	**Drug conjugated **	**Anticancer activity in cellular models **	**Therapeutic efficacy in mouse models **	**Toxicological profiles **	**Evaluation stages **	**Ref. cited **
ABBV-399 (with MMAE)	AbbVie Oncology CA, USA	ABT-700, MET, human IgG1/κ	Dipeptide, cleavable	Highly specific, ~0.5 nM/L	N/A	MMAE	Highly potent: 0.05 to 18 nM/L for cytotoxicity	Xenografts & PDXs, MET over-expressed & amplified	In primate: bone marrow, liver & digestive	Clinical, Phase II	[57, 65, 83, 84]
TR1801-ADC with PBD	Tanabe Research Laboratories USA, CA, USA	hD12, MET, humanized IgG2	Dipeptide, cleavable	Highly specific, ~0.3 nM/L	30-40% internalized after 24h	PBD	Highly potent: 0.04 to 1.3 nM/L for cytotoxicity	Xenografts & PDXs, MET over-expressed & amplified	In rat, human in progress	Clinical, Phase I	[58, 86]
SHR-A1403 (with SHR152852)	Hengrui Medicine Co Ltd, Shanghai, China	SHR-A1403, MET, humanized IgG2	MC based, noncleavable	Highly specific, ~1.8 nM/L	50-60% internalized within 2h	MMAE	Highly potent: 0.02 to 1.5 nM/L for cell proliferation	Xenografts & PDXs, MET over-expressed & amplified	In primate: bone marrow, liver & digestive	Clinical, Phase I	[59, 60, 85]
P1E2-vc-MMAF	Tanabe Research Laboratories USA CA, USA	P1E2, MET, mouse IgG	Dipeptide, cleavable	Highly specific, ~0.9 nM/L	N/A	MMAF	Highly potent: 0.01 to 0.3 nM/L for cytotoxicity	Xenografts & PDXs, MET over-expressed & amplified	In mouse only	Pre-clinical	[86]
P3D12-vc-MMAF	Tanabe Research Laboratories USA, CA, USA	P3D12, MET, mouse IgG	MC based, noncleavable	Highly specific, ~0.8 nM/L	1.0 μg/ml: ~60% MET internalized within 18h	MMAF	Highly potent: 0.05 to 26 nM/L for cytotoxicity	Xenografts & PDXs, MET over-expressed & amplified	In mouse only	Pre-clinical	[86]
c-Met-IgG-OXA, MetFab-DOX	Nanjing Medical University, Jiangshu, China	Anti-c-Met-IgG, MET, humanized IgG	Unknown	Highly specific, ~13 nM/L	IE50: 50% internalized within 3h	DOX & OXA	Moderately potent: ~11 μM/L for cytotoxicity	Xenografts, MET overexpressed	Unknown	Pre-clinical	[89, 90]
PCM-MET01-MMAE	PCM Targetech, TX, USA	PCMC1D8, MET, humanized IgG1	Dipeptide, cleavable	Highly specific, ~1.6 nM/L	IE50: 50% receptor internalized within 8.2h	MMAE	Highly potent: ~0.6 to 8.0 nM/L for cytotoxicity	Xenografts, MET overexpressed & amplified	In mouse only	Pre-clinical	[78]
HucMET27-DGN549 & HucMET27-DM4	ImmunoGen, Inc., MA, USA	HucMET27, MET humanized IgG	MC or dipeptide, cleavable & noncleavable	specific at nanomolar range	N/A	DGN549 & DM4	Highly potent at nanomolar range for cell killing	Xenografts & PDXs, MET over-expressed & amplified	Unknown	Pre-clinical	[87]
Zt/g4-DM1 & Zt/g4-MMAE	Texas Tech University HSC, TX, USA	Zt/g4, RON, humanized IgG1	MC or dipeptide, cleavable & noncleavable	Highly specific, ~3.0 nM/l	IE50: 50% receptor internalized within 15.3h	DM1, MMAE, & DCM	Highly potent: 0.5 to 25 nM/L for viability, & cell death	Xenografts & PDXs, RON over-expressed	In primates: bone marrow, liver & digestive	Pre-clinical	[61, 62]
PCM5B14-MMAE, PCM5B14-DCM	PCM Targetech, TX, USA	PCM5B14, RON, humanized IgG1	Dipeptide, cleavable	Highly specific, ~2.3 nM/L	IE50: 50% receptor internalized within 9.5h	MMAE &DCM	Highly potent: 0.5 to 25 nM/L for viability, & cell death	Xenografts, RON overexpressed	In primate: bone marrow, liver & digestive	Pre-clinical	[63]
PCMdt-MMAE	PCM Targetech, TX, USA	PCMbs-MR, MET & RON, humanized IgG1	Dipeptide, cleavable	Highly specific, ~2.5 nM/L	IE50: 50% receptor internalized within 15.7h	MMAE	Strong at nM/L in cell cycles, viability, & cell death	Xenografts, MET & RON over- expressed	In mouse only	Pre-clinical	[78]
B10v5x225-H-vc- MMAE	Technische Universität Darmstadt, Germany	B10v5x225-H, MET & EGFR, humanized IgG	Dipeptide, cleavable	Specific but affinity unknown	N/A	MMAE	Highly potent: 0.05 to 18 nM/L for cytotoxicity	Xenografts & PDXs, MET over-expressed & amplified	In mouse only	Pre-clinical	[82]
B10v5x225-M-vc- MMAE	Technische Universität Darmstadt, Germany	B10v5x225-M, MET & EGFR, humanized IgG	Dipeptide, cleavable	Specific but affinity unknown	N/A	MMAE	Highly potent: 0.05 to 18 nM/L for cytotoxicity	Xenografts & PDXs, MET over-expressed & amplified	In mouse only	Pre-clinical	[82]

^a^All information described in the Table 1 is from published articles. *ADC *Antibody-drug conjugates, *DCM *Duocarmycin, *DGN549* a DNA-alkylating payload indolinobenzodiazepine, *DM1 *Maytansinoid derivative 1, *DM4 *Maytansinoid derivative 4, *DOX *Doxorubicin, *EGFR *Epidermal growth factor receptor, *IE50* Internalization efficacy, *MC *Maleimidocaproyl, *MET *Mesenchymal-epithelial transition, *MMAE *Monomethyl auristatin E, *MMAF *Monomethyl auristatin F, *N/A *Not available, *PBD*, Pyrrolobenzodiazepines, *PDX *Patient derived tumor xenografts, *OXA *Oxaliplatin, *vc *Chemical linker containing Val-Cit structures, *SHR15852 *A synthetic auristatin analog, *RON* Recepteur d’Origine nantais

Several strategies have been used to develop TMAB-based biotherapeutics for MET and/or RON targeted therapy. The use of conventional TMABs is one approach. The lead TMABs targeting MET include ARGX-111 [64], telisotuzumab (ABT-700) [65], onartuzumab (MetMab) [66], emibetuzumab (LY2875358) [67], SAIT-301 [68], Sym015 [69], and others. TMABs targeting RON include IMC-41A10 [7-], narnatumab (NCT01119456) [71], Zt/f2 [72], and recently produced 6E6, 6D4, 7G8 [73]. In addition, bispecific TMABs targeting both MET/EGFR [74], MET/epithelial cell adhesion molecule (EpCAM) [75], MET/program cell death-1 (PD-1) [76], MET/VEGFR2 [77], and MET/RON [78] have also been reported. Some of them such as telisotuzumab, SAIT301, Sym015 are currently under clinical trials (www.clinicaltrials.gov). The rationale behind this strategy is based on the belief that cancer cells are highly addicted to MET and/or RON signaling for growth/survival. Indeed, there is evidence indicating CRAC cells that are addicted to MET and/or RON signaling yield biological consequences [11, 12]. TMAB-mediated mechanisms of action include neutralization of ligand-binding, prevention of receptor dimerization, induction of receptor internalization/degradation, attenuation of tumorigenic signalling, and stimulation of immune activities [64–78]. In animal models, both anti-MET and/or anti-RON TMABs are able to inhibit tumor growth initiated by MET and/or RON-expressing CRAC cell lines [64–78]. Nevertheless, the observed therapeutic efficacies vary significantly due to the amount of MET and/or RON expression, addictive levels to cellular signalling, and biochemical differences among individual TMABs used [64–78]. For instance, ABT-700 as a monotherapy is able to completely inhibit xenograft tumor growth [65]. In contrast, the efficacy of emibetuzumab in xenograft tumor models is relatively weak, but exhibits a strong synergistic effect with chemotherapeutics [67]. The effect of anti-RON TMABs on tumor growth, on average, is relatively weak as evident from several preclinical studies [70–73]. Complete inhibition by a single anti-RON TMAB has not been observed. These findings are not surprising because the evidence of cancer cells being addicted to MET or RON signaling for survival/growth is not a common event [11, 12]. Thus, results from these studies raise concerns about the feasibility of conventional anti-MET and/or RON TMABs for clinical application. Indeed, the discontinuation of narnatumab and onartuzumab in clinical trials is an example. Considering the fact that efficacies of anti-MET and/or anti-RON TMABs are highly dependent on cellular addiction to MET and/or RON signaling, selection of TMABs with unique pharmaceutical features appears to be important for the success of their clinical application. In this sense, the second strategy using anti-MET and anti-RON mAb-directed drug delivery in the form of an ADC, which has significant increase in the therapeutic index, is likely to be a logical step for MET and RON-targeted cancer therapy.

### ADCs Targeting MET and/or RON for CRAC Treatment

**Fig. 3:**
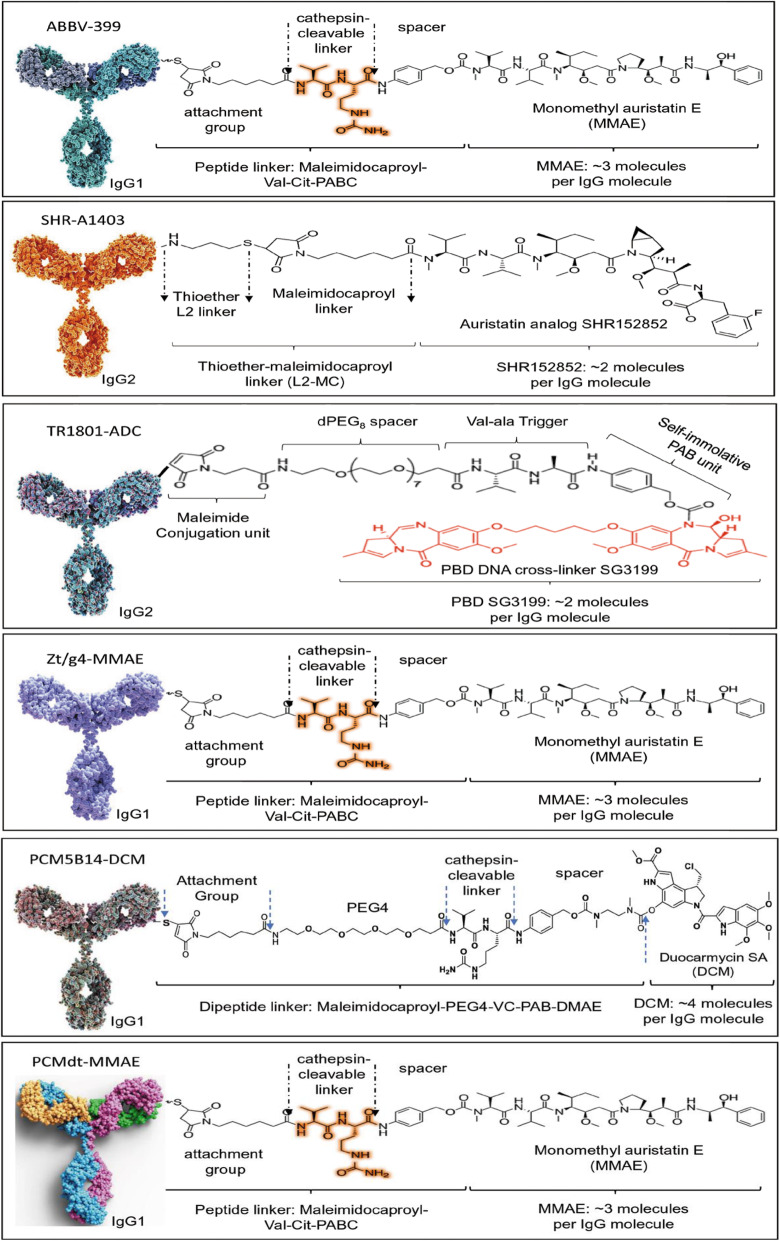
Schematic representation of anti-MET, anti-RON, MET/RON, and MET/EGFR-dual targeting ADCs. Schematic representation of anti-MET ADCs ABBV-399, SHR-A1403, and TR1801-ADC, anti-RON ADCs Zt/g4-MMAE and PCM5B14, and MET/RON dual targeting ADC PCMdt-MMAE. ABBV-399 is generated by conjugation of ABT-700 with MMAE by dipeptide cleavable linker with an average DAR of ~ 3.1. SHR-a1403 is made by conjugation of HTI-1066 with auristatin analog SHR152852 through a noncleavable linker with an average DAR of ~ 2.0. TR1801-ADC is developed by site-specific conjugation of hD13 with PBD toxin-linker tesirine with an average DAR of 2.0. Zt/g4-MMAE, PCM5B14-DCM and PCMdt-MMAE are all conjugated through dipeptide cleavable linkers

An ADC is a class of biotherapeutic that combines the specificity of antibody with a potent cytotoxin for cancer therapy. At present, seven ADCs including ado-trastuzumab emtansine, brentuximab vedotin, and sacituzumab govitecan have been approved for clinical application (www.fda.gov). Since 2013, several anti-MET mAbs including ABT-700, P3D12, and HTI-1066 have been selected for drug conjugation, resulting in several anti-MET ADCs, such as telisotuzumab vedotin (ABBV-399) [57], TR1801-ADC [58], and SHR-A1403 [59, 60] (Fig. 3; Table 1). RON-targeted ADCs such as Zt/g4-MMAE and PCM5B14-ducarmycin also have been preclinically validated (Fig. 3; Table 1) and are ready for clinical trials [61–63, 79–81]. Moreover, a novel dual targeting ADC specific to both MET and RON has been validated for clinical development (Fig. 3; Table 1) [78]. Also, dual targeting ADCs specific to both EGFR and MET, such as B10v5 × 225-H-vc-MMAE, have been reported [83]. The following section will describe these ADCs in terms of pharmaceutical properties, therapeutic efficacies, and toxicological profiles relevant to CRAC therapy.

#### ADCs targeting MET for Cancer therapy:

(A) **Telisotuzumab vedotin**: This anti-MET ADC, also known as ABBV-399, is generated by conjugation of telisotuzumab (ABT-700, human IgG1/κ) with MMAE through a cleavable dipeptide linker (Fig. 3; Table 1**)** [57]. ABT-700, derived from a mouse mAb 224G11, is capable of antagonizing MET signaling in both HGF-dependent and -independent manners and inhibits tumor growth driven by MET overexpression, amplification, or autocrine HGF stimulation [65]. Results from phase I clinical trials appear to be encouraging [83, 84]. These properties make ABT-700 a suitable candidate for ADC development. As a heterogeneous ADC with an average DAR of ~ 3, telisotuzumab vedotin has a favorable pharmacokinetic (PK) profile in the cynomolgus monkey [57]. Primary toxicities after repeated dosing in monkeys are either non-adverse or reversible [57]. Telisotuzumab vedotin *in vitro* is highly potent in killing CRAC cell lines with MET overexpression caused either by protein accumulation or by gene amplification (Table 1) [57]. An approximate threshold of cell surface MET expression (≥ 100,000 MET molecules per cell) has been established in response to telisotuzumab vedotin [57]. Cancer cells expressing low levels of MET, but have a HGF autocrine activation loop, are also sensitive to telisotuzumab vedotin. It is noticed that the effect of telisotuzumab vedotin is mainly mediated by the action of MMAE. ABT-700-mediated blockage of the MET signaling pathway in the form of an ADC is probably minimal [57]. Also, normal epithelial and endothelial cells that naturally express low levels of MET are insensitive to telisotuzumab vedotin, mainly due to the amount of MET expression falling below the threshold level required for significant killing. These findings provide the rationale explaining the minimal risk of non-target toxicity of telisotuzumab vedotin.

**Fig. 4:**
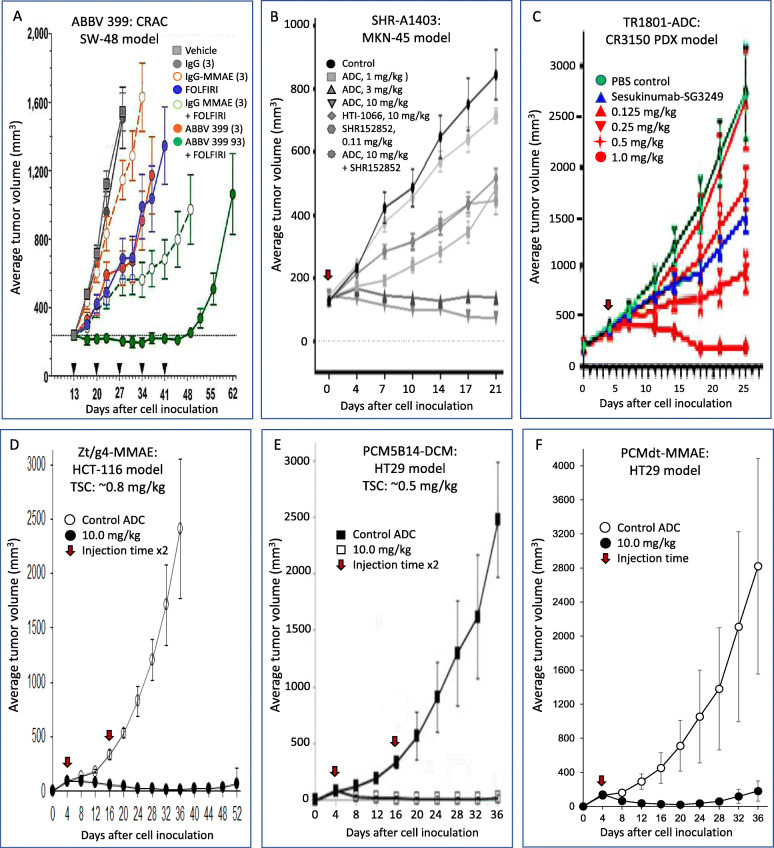
Therapeutic efficiencies of anti-MET, anti-RON, and MET/RON dual targeting ADCs in CRAC xenograft models**.** Results shown here are from published articles. CRAC xenograft tumors used are initiated by SW-48 (for ABBV-399), HCT-116 (for Zt/g4-MMAE), and HT29 (for PCM5B14-DCM) cell lines, respectively. The CR3150 PDX model is used for TR1801-ADC. The gastric tumor model initiated by MKN-45 is used for SHR-A1403. Individual ADCs are used at different doses in a different schedule or in combination with chemotherapeutics. Tumor volumes from control and ADC treated animals were measured to determine the ADC efficacy. In some cases, tumoristatic concentrations (TSCs) are calculated. It needs to be mentioned that studies shown here vary significantly with different CRAC cell lines, variable doses and treatment schedules, and with or without chemotherapeutics. Thus, results are not intent for comparison of therapeutic efficacies among individual ADCs. Instead, it is only to confirm the anticancer activity under their own doses and treatment schedules. FOLFIRI, a standard CRAC treatment regimen composed of 5-fluorouracil, irinotecan, and leucovorin; SHR152852, an auristatin analog as the payload for SHR-A1403; and SKM-SG3249, an ADC composed of the monoclonal antibody secukinumab to interleukin-17A conjugated with PBD DNA cross-linker SG3249

A series of mouse xenograft models including patient-derived xenografts (PDX) and tumors refractory to MET-specific SMKIs have been used to evaluate the efficacy of telisotuzumab vedotin (Fig. 4a) [57]. In all xenograft tumor models including PDXs tested, telisotuzumab at 1 to 3 mg/kg in a Q4 × 6 schedule is highly effective with complete and durable tumor regression. Significantly, telisotuzumab vedotin is more effective for both MET-overexpressed and amplified tumors than those with low to moderate levels of MET expression. Telisotuzumab vedotin shows synergistic activities with chemotherapeutics. For instance, telisotuzumab vedotin or FOLFIRI (a three-drug regimen: 5-fluorouracil, leucovorin, and irinotecan) as monotherapy only partially inhibits CRAC xenografts mediated by SW-48 cells expressing low levels of MET. However, telisotuzumab vedotin in combination with FOLFIRI results in a significant increase in therapeutic efficacy [57]. This finding provides the rationale for clinical CRAC application using telisotuzumab vedotin based chemo-combination therapy. Another feature is that telisotuzumab vedotin is effective in xenograft tumors refractory to the action of ABT-700. As described above, ABT-700 has a direct effect on tumor growth but eventually results in acquired resistance due to repeated exposures [65]. Treatment of these tumors with telisotuzumab vedotin at 3 mg/kg in a Q4 × 6 schedule is sufficient to cause a durable regression for these ABT-700 refractory tumors [57]. These data indicate that tumors insensitive to MET pathway inhibition remain sensitive to telisotuzumab vedotin. In conclusion, telisotuzumab vedotin has the potential as an effective biotherapeutic in MET-targeted cancer therapy.

Phase I clinical trials of telisotuzumab vedotin, that ended in the middle of 2018, show promising results for this ADC [83]. Among 48 patients with advanced solid cancers evaluated, telisotuzumab displays a favorable PK profile with a mean harmonic half-life of 2 to 4 days. Telisotuzumab vedotin is relatively safe with acceptable adverse events mostly reported as fatigue, gastrointestinal symptoms, peripheral edema, and neuropathy. Less than 4% of patients developed grade ≥ 3 adverse events such as anemia, neutropenia, and hypoalbuminemia. Three out of 16 NSCLC patients with MET expression achieved a partial response after treatment of telisotuzumab vedotin up to 3.0 mg/kg. The observed median response duration was reported as 4.8 months, the median progression-free survival as 5.7 months, and the 95% confidence intervals as 1.2 months to 15.4 months [83]. These observations warrant the transition of telisotuzumab vedotin into the phase II clinical trials.

B **SHR-A1403**: This ADC is formed by humanized anti-MET mAb HTI-1066 (also known as SHR-A1403 mAb, IgG2) with payload SHR152852 using a non-cleavable linker (Fig. 3; Table 1) [59, 60]. The resulting ADC is heterogeneous and currently under phase I clinical trials (NCT03856541). Humanized HTI-1066 binds to MET from human and monkey species with high affinities, interacts at very low affinities with cellular Fc receptors and complement component C1q, and strongly induces MET internalization [59, 60]. SHR152852 is a microtubule inhibitor derived from a series of chemically designed auristatin analogs [59, 60]. The rationale to select SHR152852 was to have a payload with reduced cytotoxicity for liver cells in free drug form but sustained activity in the form of an ADC for cancer cell killing [59, 60]. The resulting SHR-A1403 ADC, having a DAR of 2, has a favorable PK profile in animals including the cynomolgus monkey with minimal dissociation *in vivo*. In mice bearing xenografts expressing MET, exposure to SHR-A1403 is proportional to the dose administrated with a low clearance (CL: 0.58–0.78 ml/h/kg) and a relatively long terminal half-life (t½: 6.1 day) [85]. Similar PK profiles are also observed when rats are used as the model [59, 60, 85]. The PK profile of SHR-A1403 in the cynomolgus monkey shows a non-linear behavior with an average t½ at 5.5 days and CL at 0.39–0.822 ml/h/kg. Serum concentrations of the free toxin are below the limit of quantitation,^85^ indicating low systemic exposure of the free toxin to tissues.

Functionally, SHR-A1403 *in vitro* strongly causes cell cycle arrest and inhibits proliferation of cancer cells from gastric, lung, breast, prostate, renal, and hepatic tissues [59, 60]. SHR-A1403 also inhibits NSCLC cell lines resistant to EGFR inhibitor AZD9291 with or without MET phosphorylation [59, 60]. The therapeutic activity of SHR-A1403 *in vivo* has been determined in several xenograft tumor models using cancer cell lines derived from liver, lung, and gastric tissues with different malignant and chemoresistant statuses and of hepatocellular carcinoma (HCC) PDX models (Fig. 4b). The obtained results show that SHR-A1403 effectively delays xenograft tumor growth in all models tested with MET overexpression, regardless the origin of cancer cells [59, 60]. The effect appears to be mediated by cytotoxic payload but not by antibody antagonist activity or immune reactivity. The involvement of immune response appears to be minimal. Nevertheless, tumor eradication from all models evaluated has not been demonstrated [59, 60]. In addition, SHR-A1403-mediated tumor growth inhibition is in a dose-dependent manner and long-lasting. Complete growth inhibition is mostly observed when the ADC is used at 10 mg/kg in a single injection schedule [59, 60]. SHR-A1403 at 3 mg/kg only partially delays tumor growth without completely controlling xenograft growth. Moreover, SHR-A1403 at 1 mg/kg, in general, is ineffective or exhibits marginal activities [59, 60]. Calculation of the minimal doses required to balance the tumor growth and inhibition from three models has revealed that SHR-A1403 has a TSC around ~ 0.35 mg/kg [59, 60]. Considering these facts, SHR-A1403 in a once every 3-week dosing regimen has been selected for phase I clinical trials. At present, data from clinical trials are not available.

C **TR1801-ADC**: This “third generation” ADC is developed through site-specific conjugation of humanized mAb hD12 (IgG2) to PBD toxin-linker tesirine (Fig. 3; **Table 1**) [58]. TR1801 is currently in phase I clinical trials (NCT03859752). The anti-MET hD12 is derived from mouse anti-MET mAb P3D12 (IgG1) and was based on its strong binding affinity, robust induction of receptor internalization, and minimal agonistic activities [86]. P3D12 and another anti-MET mAb P1E2 have been conjugated with MMAF to form ADCs P3D12-vc-MMAF and P1E2-vc-MMAF, which have superior potencies in MET amplified and non-amplified cancer models [86]. Modification of P3D12 through humanization, IgG subclass switching, and site-specific cysteine incorporation results in hD12 for TR1801-ADC development [58]. The PBD toxin-linker tesirine, known as SG3249 developed by Spirogen, is featured by straightforward cysteine conjugation, good solubility in aqueous/DMSO, and a versatile cleavable linker for delivering PBD DNA cross-linker SG3199 [55]. The use of PBD in ADCs has been in pharmaceutical interests. PBD is a class of sequence-dependent DNA alkylating compounds derived from various actinomycetes and proven to be more powerful than systemic chemotherapeutic drugs [55]. These features make PBD an excellent payload in ADCs. The rationale of selecting SG3249 for site-specific hD12 conjugation instead of using MMAF as the payload appears to be based on the fact that using low doses of antibody in combination with a highly potent cytotoxin is less likely to develop resistance but capable of achieving a maximal therapeutic index with acceptable tolerability and manageable toxicities in MET-targeted cancer therapy.

TR1801-ADC is homogeneous with an average DAR of ~ 2.0 [58]. Toxic studies in rats reveal that TR1801-ADC is well tolerated up to 2 mg/kg with no significant bodyweight reduction or clinical abnormalities [58]. TR1801-ADC *in vitro* is highly potent against cancer cells expressing high levels of MET (~ 100,000 to ~ 450,000 MET molecules per cell) with or without MET gene amplification (**Table 1**). Also, CRAC cell lines expressing low to moderate levels of MET (5,000 to 90,000 MET receptor molecules per cell) are sensitive to TR1801-ADC [58]. In a comparative analysis, TR1801-ADC is far more efficient than P3D12-vc-MMAF and another anti-MET ADC MET-vc-MMAE in killing MET expressing cancer cells [58, 86]. It appears from the *in vitro* studies that among all anti-MET ACDs tested, TR1801-ADC is the most potent in killing cancer cells expressing variable levels of MET.

Both *ex vivo* 3D tumoroid and xenograft tumor models including primary PDXs have been applied to validate TR1801-ADC (Fig. 4c) [58]. Cancer cell lines used include those from lung, gastric, colorectal, and head & neck tissues. TR1801-ADC is used at 0.125 mg/kg to 1 mg/kg in a single injection schedule. In general, TR1801-ADC is highly effective in inhibition of tumor growth in both *ex vivo* 3D and xenograft models regardless of the source of cancer cell lines used with moderate to high levels of MET expression [58]. In certain gastric PDX models, TR1801-ADC at 0.125 mg/kg in a single dose injection still shows significant growth inhibition with durable activity [58]. In CRAC PDX models, TR1801-ADC shows significant growth inhibition in 9 out of 10 cases. Complete tumor inhibition is documented in 4/10 (40%) cases [58]. The effective doses of TR1801-ADC are around 0.5 to 1 mg/kg in a single dose-injection schedule. Activities from 0.125 to 0.25 mg/kg appear to be weak with only ~ 10 to ~ 30% growth inhibition, dependent on individual PDX cases tested. Importantly, levels of MET expression appear to be positively correlated with the efficacy of TR1801-ADC. For instance, the PDX model CR3150 with MET overexpression shows to be more responsive to a single dose of TR1801-ADC than the model CR0126 that shows moderate MET expression [58]. It is worth mentioning that the efficacy of TR1801-ADC in CRAC PDX models is long-lasting. In tumors with moderate to high levels of MET expression, a single dose of TR1801 at 1 mg/kg is sufficient to inhibit tumor growth up to 4 weeks without signs of tumor regrowth. Nevertheless, it is noticed that TR1801-ADC did not eradicate tumors in all models tested [58].

D **HucMet27-based ADCs**: HucMet27 was generated by Immunogen in 2017 as a humanized anti-MET IgG mAb with minimal agonistic activity [87]. The objective was to use hucMet27 to develop a MET-targeted ADC for tumors harboring MET overexpression and/or amplification. The payload for hucMet27 is a highly potent indolinobenzodiazepine DNA-alkylating payload (DGN549), which is conjugated to hucMet27 through a site-specific conjugation method, resulting in anti-MET ADC hucMet27-DGN549 [87]. HucMet27 has also been conjugated with maytansine derivative DM4 using a N-succinimidyl-4-(2-pyridyldithio)-2-sulfo butanoate (sulfo-SPDB) linker to generate hucMet27-DM4 [87]. Studies *in vitro* demonstrate that hucMet27-DGN549 exerts strong cytotoxicity against a large panel of MET-expressing cancer cell lines. By contrast, the potency of hucMet27-DM4 is restricted mainly to cell lines harboring MET amplification, despite all cell lines demonstrating sensitivity to the unconjugated payload [87]. In mice bearing xenograft tumors, both hucMet27-DGN549 and hucMet27-DM4 are highly effective in MET-amplified models. Interestingly, hucMet27-DGN549 was more potent in induction of tumor regressions in the model with MET overexpression without MET amplification [87]. These results indicate that hucMet27-DGN549 is a good candidate with potential application in MET-targeted cancer therapy. At present, hucMet27-based ADCs appear to still be in the preclinical stage.

#### ADCs targeting RON for potential cancer therapy:

(A) **Antibodies selected for ADC development**: Currently, two anti-RON mAbs, Zt/g4 and PCM5B14, have been selected, resulting in Zt/g4- and PCM5B14-based ADCs for potential clinical evaluation (Fig. 3) [61–63, 79–81]. Both mAbs are specific to the RON extracellular domains. Zt/g4 strongly interacts with the RON semaphorin (SEMA) domain and PCM5B14 recognizes the RON plexin-semaphorin-integrin (PSI) domain [61–83]. They also bind to monkey RON with similar binding affinities but not to canine or mouse RON homologues [61–83]. Both mAbs induce a robust RON internalization by CRAC cells, which ensures sufficient amounts of payload delivered for cytotoxic activity and avoids potential development of chemoresistance. Both mAbs have been conjugated with different payloads such as DM1, MMAE, and DCM with proper conjugation profiles, serum stability, and PK parameters [61–63, 79–81]. These features make Zt/g4 and PCM5B14 ideal candidates for anti-RON ADC development.
(B) **Cytotoxicity of anti-RON ADCs in CRAC cellular models**: Various CRAC cell lines with different subtypes, malignant status, drug sensitivity, and levels of RON expression have been tested for their response to anti-RON ADCs [61–63]. Both Zt/g4- and PCM5B14-based ADCs significantly arrest CRAC cell cycle, reduce cell viability, and cause massive cell death. The effectiveness of the ADCs is proportionally correlated with the level of RON expression by CRAC cells. A minimal of 8,000 RON receptors per cell appears to be required for anti-RON ADCs to achieve a 95% reduction in cancer cell viability [61–63, 79–81]. The use of establishing minimal RON expression needed for reduction of cell viability as a threshold to achieve maximal activity may have potential to serve as a reference in selecting CRAC patients for anti-RON ADC therapy in clinical evaluation.
(C) **Therapeutic efficacy of anti-RON ADCs in CRAC xenograft models**: Studies from animal models have proven the efficacy of both Zt/g4- and PCM5B14-based anti-RON ADCs in inhibition and/or eradication of CRAC cell-derived xenograft tumors (Fig. 4d and e) [61–63, 79–81]. **First**, all anti-RON ADCs including Zt/g4-DM1, Zt/g4-MMAE, PCM5B14-MMAE, and PCM5B14-DCM in a single-dose treatment regimen are highly effective against CRAC xenografts mediated by different CRAC cell lines with different phenotypes. Results from dose-dependent treatment also confirms the superiority of both Zt/g4- and PCM5B14-based ADCs against CRAC xenograft tumors. The calculated TSCs are ~ 5.0 mg/kg for Zt/g4-DM1, ~ 1.5 mg/kg for Zt/g4-MMAE, ~ 1.3 mg/kg for PCM5B14-MMAE, and ~ 0.3 mg/kg for PCM5B14-DCM [61–63]. These values are in line with the doses of ADCs currently approved by the FDA for clinical application. **Second**, both MMAE and DCM-conjugated ADCs not only inhibit tumor growth but also eradicate tumors at variable degrees regardless of their chemoresistant or metastatic status [61–63, 79–81]. However, the DM1-based anti-RON ADC, Zt/g4-DM1, exerts only tumor-inhibitory but not eradicating activities [61–63]. Considering these observations, MMAE-based anti-RON ADCs are favored as the lead candidates for clinical evaluation. **Third**, MMAE-based ADCs have the ability to kill RON-negative CRAC cells through bystander mechanisms [61–63], which may explain the ability of MMAE-based ADCs, but not DM1-based ADCs, in eradicating tumor xenografts. **Finally**, the presence of residual tumors with minimal RON expression after anti-RON ADC treatment suggests a necessity in combination with chemotherapeutics or immunoregulatory agents to prevent tumor recurrence. In this sense, anti-RON ADCs in combination with chemotherapeutics, SMKIs, immune checkpoint antibodies, or others should be considered for clinical application.
(D) **Pharmacokinetic and toxicological Features of Anti-RON ADCs**: Both Zt/g4 and PCM5B14 based ADCs, formed either by noncleavable or by cleavable linkers, are stable in human plasma up to 30 days with less than 4.0% of payloads dissociated from the antibody [61–63, 79–81]. Results from the cynomolgus monkey confirm this finding, suggesting that anti-RON ADCs are highly stable *in vivo* [61–63]. The PK profiles of Zt/g4-MMAE in the cynomolgus monkey fit the two-compartment model [62]. Zt/g4-MMAE has an average mean plasma clearance of 0.12 ml/day/kg, a t½ of ~ 6.5 days, and a mean residential time of ~ 7.50 days in the cynomolgus monkey [62]. These observations demonstrate that anti-RON ADCs are stable and display a favorable PK profile.

Toxicological analysis in both mice and cynomolgus monkeys demonstrate that anti-RON ADCs are safe with manageable adverse activities [61–63, 79–81]. The maximal tolerance dose (MTD) for both MMAE- and DCM-based ADCs is ~ 60 mg/kg as judged by mouse daily activity, food consumption, and bodyweight. In monkeys, Zt/g4-MMAE at a single dose of 10 or 30 mg/kg does not cause clinical abnormalities judging from animal daily activity, bodyweight, body temperature, food consumption, heart rate, breath, vision, and urination [62]. Also, no evidence of tissue inflammation, cell death, structural alteration, hemorrhage, or other pathological changes has been observed in all animals tested [62]. Nevertheless, adverse reactions from blood chemistry analysis indicate slight to moderate abnormalities in blood leukocytes, reticulocytes, and a panel of liver enzymatic activities [62]. These effects are in a dose-dependent manner, reversible, and manageable. At the end of the study, all changes were restored to the baseline. It is noticed that toxicological profiles of Zt/g4-MMAE are highly similar to those of ADCs approved by the FDA or those currently under clinical trials [88]. Specifically, toxicities of ADCs conjugated with MMAE all cause similar reactions in the hematopoietic system, liver, and reproductive system regardless of antibodies used [88]. The toxic effect of DCM-based anti-RON ADCs appears to be severe in the cynomolgus monkey (our unpublished data). PCM5B14-DCM at a single injection of 30 mg/kg leads to death of animals (our unpublished data). These data help to design phase 1 clinical trials for Zt/g4- and PCM5B14-based ADCs.

#### Dual-targeting ADCs specific to both MET and RON

**(A) MET and RON dual targeting ADC**: PCMdt-MMAE is the first dual targeting ADC specific to both MET and RON (Fig. 3; Table 1) [78]. Developed by the PCM Targetech LLC in 2020, the humanized bispecific antibody PCMbs-MR (IgG1/κ) is generated by grafting sequences from both anti-MET mAb PCM-MET01 and anti-RON mAb PCM-5B14 into the human IgG1 backbones. Modifications and optimizations were performed to ensure the proper formation of bispecific IgG molecules. PCMbs-MR has several features suitable for development of a dual-targeting ADC. **First**, PCMbs-MR recognizes MET and RON from both human and monkey but not those from canine or mouse [78]. The dual antigen-binding specificity is inherited from PCM-MET01 and PCM5B14 [63, 78]. **Second**, PCMbs-MR has the ability to rapidly induce both MET and RON internalization. The observed internalization efficacies (IE50) range from 10 to 20 h dependent on cellular levels of MET and RON expression, which results in delivery of sufficient amounts of payload for cancer cell killing [78]. **Third**, PCMbs-MR is suitable for conjugation with various payloads, including MMAE and DM-1, using either cleavable or noncleavable linkers. The resulting ADCs are stable in serum and have a favorable PK profile in mice [78]. **Finally**, the fact that PCMbs-MR recognizes not only human MET and RON but also their monkey corresponding homologies make it suitable for using the monkey model to study the PK profile and toxicological activity of PCMdt-MMAE.

The biological activities of PCMdt-MMAE *in vitro* meet pharmaceutical expectations in terms of its potency in killing CRAC cells. Flow cytometric analysis indicates that PCMdt-MMAE causes cell cycle arrest in the G2/M phase. This effect is observed as early as 12 h after ADC treatment and characterized by progressive reduction of the G1 phase and the accumulation of cells at the G2/M phase [78]. PCMdt-MMAE also decreases cell viability in a dose-dependent manner in a panel of CRAC cell lines expressing variable levels of MET and RON. More than a 60 to 80% reduction in cell viability 96 h after ADC treatment is achieved among CRAC cancer cell lines tested. Importantly, PCMdt-MMAE induces a massive cell death in various CRAC cell lines in a dose-dependent manner with IC_50_ values ranging from 1 to 15 nM. Analysis of cellular morphology and apoptotic markers has confirmed PCMdt-MMAE-induced CRAC cell death [78]. The fact that cell death was minimal in cells lacking MET and RON expression suggests that the action of PCMdt-MMAE is mediated through a target-specific manner.

Studies from mouse models prove the therapeutic effectiveness of PCMdt-MMAE (Fig. 4F). This conclusion is supported by three sets of experiments. **First**, in a comparative study using anti-MET ADC PCM-MET01-MMAE and anti-RON ADC PCM5B14-MMAE for comparison, PCMdt-MMAE on average reduces tumor volume up to 93%, decreases tumor weight up to 96%, and eradicates tumors up to 20%. The calculated TSC for PCMdt-MMAE is 0.35 mg/kg, similar to that for PCM-MET01-MMAE and PCM5B14-MMAE (0.51 mg/kg and 0.32 mg/kg. respectively) [78]. These results confirm that the therapeutic efficacy of PCMdt-MMAE is comparable to that of PCM-MET01-MMAE and PCM5B14-MMAE. **Second**, in a concentration-dependent experiment, PCMdt-MMAE at 1 mg/kg is sufficient to inhibit CRAC xenograft growth and prevent tumor regrowth for up to two weeks. Additional analysis confirms reduction of tumor weight ranging from 65–99% dependent on the dose of PCMdt-MMAE used. Also, PCMdt-MMAE at 3 to 10 mg/kg is capable of eliminating tumors at variable levels [78]. Thus, PCMdt-MMAE not only inhibits tumor growth but also eradicates xenografts when used at relatively high therapeutic doses. **Third**, in tumors mediated by cancer cell lines from colon, lung, pancreas and breast, regardless of their metastatic and chemoresistant status, PCMdt-MMAE shows a broad anticancer activity leading to inhibition of all these xenografts. The inhibition by average tumor volume was 97% for CRAC HCT116, 86% for SCLCS H358, and 91% for pancreatic cancer BxPC-3 mediated tumors. More importantly, the effect of PCMdt-MMAE is long-lasting. At 10 mg/kg in a single dose injection schedule, PCMdt-MMAE inhibits xenograft tumor growth for almost four weeks, equivalent to a ~ 6 half-life cycle *in vivo* [78]. Thus, PCMdt-MMAE has superior anticancer activity, which warrants its transition into clinical trials in the near future.

The use of mouse models for the PK profiling provides insight into the dynamics of PCMdt-MMAE *in vivo.* It appears that the PK profile of PCMdt-MMAE in mice displays a two-compartment model, similar to other clinically approved ADCs such as T-DM1. Moreover, the PK profiles of PCMdt-MMAE between tumor-bearing and -nonbearing mice show no differences. Overall, data from tumor-bearing mice overlap with those from tumor-nonbearing mice with 95% prediction intervals [78]. Additional discovery is that overexpression of MET and RON in xenograft tumors has no impact on the fate of PCMdt-MMAE *in vivo*. In other words, tumors constitutively expressing MET and RON probably have very little effect on absorption, distribution, metabolism, and excretion of PCMdt-MMAE [78]. Nonetheless, since PCMdt-MMAE does not recognize the mouse homolog of MET and RON expressed by various tissues/organs, a PK profile of PCMdt-MMAE in human subjects should determine whether normal tissues/organs expressing low levels of MET or RON affect the PK profile of PCMdt-MMAE. Regardless of these considerations, PCMdt-MMAE has a favorable PK profile, which provides the pharmaceutical basis in clinical trials to determine its therapeutic efficacy.

Toxic activities of PCMdt-MMAE at therapeutic doses in mice appears to be safe with minimal impact on animal’s behavior or body weight. However, a single dose of PCMdt-MMAE at 30 mg/kg has a negative impact in mice highlighted by slight changes in mouse behavior and by moderate reduction in bodyweight for a short period [78]. This implies that during multiple administrations of PCMdt-MMAE for cancer treatment, doses accumulated *in vivo* should not exceed 30 mg/kg limitation. Considering the average TSCs (0.35 mg/kg) of PCMdt-MMAE in xenograft models tested, the dose limitation judged by mouse bodyweight should be a valuable reference, together with toxicological studies in primates, for the use of PCMdt-MMAE in a first-in-human study in the future.

**(B) MET and EGFR dual targeting ADC**: At present, two dual targeting ADCs specific to both MET and EGFR, namely B10v5 × 225-H-vc-MMAE and B10v5 × 225-M-vc-MMAE, have been described (Table 1) [82]. The optimized sequences specific to the EGFR epitope with moderate and high binding affinity, known as 225-M, and 225-H, are derived from cetuximab (C225). The optimized sequences specific to the MET SEMA domain (B10v5) were from a phage displayed anti-MET antibody B10 [82]. The objective was to develop bispecific antibodies with potentially increased tumor selectivity through affinity-attenuated variants while decreasing cytotoxic effects on normal cells. Through a strand exchange engineered domain technology, bispecific antibodies B10v5 × 225-M and B10v5 × 225-H showing appropriated binding affinities to both MET and EGFR have been generated with early stages of preclinical validation [82]. Studies from cellular models show that both B10v5 × 225-M and B10v5 × 225-H retain capability to block ligand-induced receptor activation of MET and EGFR, which results in inhibition of MET signaling and EGFR phosphorylation. Both antibodies also cause robust internalization of cell surface MET and EGFR. Furthermore, these bispecific antibodies are able to induce ADCC [82]. These features make B10v5 × 225-M and B10v5 × 225-H suitable candidates for generation of bispecific ADCs, leading to the formation of two MET/EGFR dual targeting ADCs, B10v5-x225-M-vc-MMAE and B10v5 × 225-H-vc-MMAE, with a DAR of 2 [82]. Cytotoxic analysis using a panel of cancer cell lines with different combinations of MET and EGFR expression show that both dual targeting ADCs are effective in killing cancer cells with IC_50_ values ranging from 0.4 to 1.0 nM/L [82]. Unfortunately, the efficacy of these two dual-targeting ADCs in xenograft tumor models has not been reported. From the conceptual point of view, studies described above demonstrate the feasibility and potential of MET/EGFR dual targeting ADCs for targeted cancer application.

## Conclusions and perspectives

Aberrant MET and RON expression and signaling are malignant features in CRAC with different behaviors and phenotypes. Altered expression of MET, RON and both receptors also have prognostic values for disease progression and patient survival. Establishment of MET and RON as therapeutic targets has led to the development of antibody-based biotherapeutics including ADCs for potential clinical application. Unlike MET-specific SMKIs that require cancer cell signaling addiction for growth and survival to show therapeutic activity, ADCs specifically deliver highly potent cytotoxic payloads for cancer cell killing and eradication. Currently, various ADCs targeting MET, RON, or both receptors are in preclinical development and some of them have advanced into clinical trials with promising results. Considering therapeutic superiorities with favorable pharmacological profiles and manageable adverse activities, ADCs targeting MET, RON, or both receptors hold the promise as an effective modality for CRAC treatment in the future. In this sense, the success of ADCs will depend on stratification of CRAC patients selected for clinical trials, suitability of optimized ADC dosing and schedule, and criteria for objective efficacy evaluation. Moreover, the advancements in IgG recombination technology, generation of versatile chemical likers, selection of suitable payloads for maximizing killing of cancer cells, and pharmaceutical improvements will lead to the development of new generations of ADCs in the near future for clinical CRAC application.

## Abbreviations

ADC: antibody-drugconjugate
APC: Adenomatous polyposis coli
BRAF: v-raf murine sarcoma viral oncogene homolog B1CRAC; colorectal adenocarcinoma
DAR: Drug to antibody ratio
DCM: Duocarmycin SA
DM1: Maytansinoidderivative 1
EMT: Epithelial-mesenchymaltransition
IHC: Immunohistochemical
IPT: Immunoglobulin-likeplexin and transcription
mAb: Monoclonal antibodies
MAP: Mitogen-activatedprotein
MET: Mesenchymal-epithelialtransition
MMAE: Monomethyl auristatinE
MMAF: Monomethyl auristatinF
PBD: Pyrrolobenzodiazepine
PK: Pharmacokinetic
PI3K: Phosphatidylinositol 3 kinase
PSI: Plexin-semaphorin-integrin
RON: Recepteur d’Originenantais
SEMA: Semaphorin
SMKI: Small molecule kinaseinhibitor
TMAB: Therapeutic monoclonalantibody
